# Development and validation of PozQoL: a scale to assess quality of life of PLHIV

**DOI:** 10.1186/s12889-018-5433-6

**Published:** 2018-04-20

**Authors:** Graham Brown, Gosia Mikołajczak, Anthony Lyons, Jennifer Power, Fraser Drummond, Aaron Cogle, Brent Allan, Craig Cooper, Simon O’Connor

**Affiliations:** 1 Australian Research Centre in Sex, Health and Society, La Trobe University, Building NR6, Bundoora, VIC 3086 Australia; 2 ViiV Healthcare Australia, 4/436 Johnston St, Abbotsford, VIC 3067 Australia; 3 National Association of People with HIV Australia, Suite G5 1 Erskineville Rd, Newtown, NSW 2042 Australia; 4 International Council of AIDS Service Organizations, 120 Carlton St, Suite 311, Toronto, ON M5A 4K2 Canada; 5 Australian Society for HIV, Viral Hepatitis and Sexual Health Medicine, Level 7, 46-56 Kippax Street, Surry Hills, NSW 2010 Australia; 6 Positive Life NSW, 414 Elisabeth St, Surry Hills, NSW Australia; 7 Queensland Positive People, 21 Manilla St, East Brisbane, QLD 4169 Australia; 8 Formerly: Living Positive Victoria, 1/111 Coventry St, Southbank, VIC 3006 Australia

**Keywords:** Quality of life, HIV, Living with HIV, Scale development

## Abstract

**Background:**

Advances in medical treatment for HIV are driving major changes in HIV policy and practice, including the encouragement of intake and adherence to HIV antiretroviral treatment (ART) by people living with HIV (PLHIV) for both personal and public health benefits. However, there is increasing recognition that achieving these goals will require a concurrent focus on the broader psychological and social wellbeing of PLHIV. Increasingly calls are being been made to incorporate a stronger focus on quality of life (QoL) of PLHIV into HIV prevention policy.

In order to achieve this goal, HIV community, support and healthcare services need a valid, short and practical way to evaluate QoL of PLHIV accessing their programs. Current QoL measures are either long, complex, restricted in their use, or expensive. To address these shortcomings, the PozQoL study aimed to develop, test and validate a short and freely available scale assessing QoL among PLHIV.

**Methods:**

Drawing on a literature review, the prioritisation of domains and development of the initial pool of items was conducted in consultation with PLHIV community organisations in Australia. The items covered health concerns, psychological, social, and functional wellbeing. Testing involved a baseline and a follow-up survey of 465 adult Australians living with HIV. Participants were recruited through social media and various community organizations nationwide. The survey included the pilot PozQoL scale and other validated measures of health and wellbeing.

**Results:**

Guided by an Exploratory Factor Analysis and conceptual considerations, a 13-item scale was developed. The PozQoL scale demonstrated high levels of fit in a Confirmatory Factor Analysis, very good internal consistency, test-retest reliability, and concurrent validity with other measures that approximated different aspects of QoL.

**Conclusion:**

The PozQoL scale has been tested in a diverse sample of adult PLHIV living in Australia, demonstrating very good reliability and validity. The insights from PLHIV and other stakeholders supported the balancing of statistical rigour and conceptual accuracy. The scale is now ready to be implemented and field-tested across a range of community, support and healthcare programs for PLHIV. This will make a significant contribution to the evaluation and enhancement of programs for PLHIV.

## Background

Health services, community organisations, and policymakers are currently responding to the largest changes in addressing HIV treatment and prevention for decades. Recent research has demonstrated the effectiveness of modern antiretroviral treatment (ART) in achieving suppression of HIV, minimising damage to the immune system while reducing the risk of onward HIV transmission nearly to zero [1]. These advances in medical treatment are changing the experience of living with HIV, and people living with HIV (PLHIV) are encouraged to seek and adhere to treatment for both personal and public health benefits. This has led to major changes in HIV policy and practice. Encouraging uptake and maintenance of ART by HIV positive people is now a central plank of national and international HIV prevention frameworks.

Since 2014, UNAIDS and the World Health Organisation have championed the 90–90-90 goals striving for 90% of all PLHIV to know their HIV status, 90% of those diagnosed to receive sustained ART, and 90% of those receiving ART to be virally suppressed by 2020 [2, 3]. Yet, there is a growing understanding that achieving these goals will require a concurrent focus on the broader psychological and social wellbeing of PLHIV, both as a means and an end goal. For example, previous evidence indicates that PLHIV with poor mental health or experiencing stigma are less likely to adhere to ART [4–7]. On the other hand, while improving health outcomes, viral suppression in itself does not ensure improved quality of life (QoL) among PLHIV [7]. For this reason, calls have been made to incorporate a stronger focus on QoL into HIV prevention and care policy. Lazarus and colleagues [8], for instance, have argued for the fourth 90 – achieving the goal of 90% of PLHIV reporting good QoL.

Recognizing the importance of QoL for PLHIV, Australian government included improving QoL of PLHIV among one of the six objectives of the Seventh National HIV Strategy 2014–2017 [9]. Following recommendations spelled out in the strategy, many community, health and policy organisations in Australia have subsequently prioritised improving the QoL of PLHIV in their programs.

Monitoring QoL among PLHIV posits however some conceptual and practical challenges. First of all, there is no consensus as to what QoL is and what key indicators constitute QoL [10]. In addition, according to different existing definitions, QoL covers a range of domains, such as physical and mental health, or subjective life satisfaction. Thus, it cannot be measured with simple measures assessing just one of these elements.

To address this complexity, several existing health-related quality of life (HRQoL) measures have been validated for PLHIV [11]. These incorporate constructs in generalised HRQoL measures, such as self-rated physical and mental health and wellbeing, as well as items specific to PLHIV, such as the impact of stigma, treatments and HIV symptomatology [11, 12]. However, past reviews [13, 14] and our analysis have identified that each of the existing scales has some limitations. Some scales (e.g., HUI3 [15], EQ-5D [16]) focus too narrowly on perceived health, neglecting other important aspects of QoL such as psychological or social wellbeing. Other scales are modified versions of existing generic scales (e.g., MOS-HIV [17]), were originally developed for patients with other conditions (e.g., FAHI; [18]), or were developed prior to the introduction of HAART (e.g., HIV-HOPES [19]), thus might not capture well aspects unique to current experiences of PLHIV. Similarly, the existing short generic QoL scales (e.g., SF-12 [20], AQoL [21], Eurohis QoL [22]) do not tap into all relevant aspects of QoL of PLHIV, particularly issues of social isolation, resilience, confidence in the future, and HIV related stigma, which many community and health programs try to address. The more comprehensive scales developed specifically for PLHIV, on the other hand, are rather long (e.g., HIV-HOPES [19], FAHI [18], PROQOL-HIV [12]) and therefore take a substantial amount of time to complete and are often too large to be incorporated into short surveys or evaluation forms. Finally, use of some scales involves licensing costs or copyright restrictions, which are often insurmountable barriers to community-run organizations. To sum up, none of the existing scales fully meet the needs of the HIV sector. In consequence, QoL instruments are rarely used in the HIV sector for program evaluation or monitoring of QoL within day-to-day health and community service practice.

### PozQoL study

HIV community, support, and healthcare services in Australia expressed a need for an empirically validated, short and practical way to measure QoL of PLHIV accessing their programs. To meet this need, the PozQoL study aimed to develop, test and validate a short and freely available scale assessing the HRQoL among PLHIV that could be easily incorporated into the day-to-day practice of health and community services as well as other community-led social research.

## Method

### Development of PozQoL

The PozQoL scale was developed in four stages: a review of the existing literature, conceptualization, item development, and validation. As with many countries, the Australian response to HIV is characterised by an active partnership between PLHIV, the community sector, researchers, clinicians and government [9]. To maintain this commitment and to deepen the understanding of QoL of PLHIV, the PozQoL study was conducted using an approach embedded in the Greater Involvement of People with HIV/AIDS (GIPA) principles [23] of a direct partnership with peer-led organisations representing PLHIV. The term Poz is a colloquial term used in a number of western countries to refer to HIV Positive people in the context of empowerment and self-determination. The term is used primarily by peer based PLHIV organisations, including those involved in this study. It was incorporated into PozQoL to reflect the strong PLHIV leadership within the study. Peer-led organisations were involved in all aspects of the study, including the conceptualisation and prioritisation of the domains, development of items, and decisions concerning the refinement of the final scale. The aim was to construct a scale that would be easy to administer and comprehensive, but also limited to no more than 15 items.

### Conceptualisation of QoL and assessment of domains

A structured literature search was conducted on relevant databases (Google Scholar, PubMed, Web of Science) to identify key definitions and domains of HRQoL of PLHIV. Keywords included “quality of life” in combination with “HIV” or “AIDS”. Preference was given to more recent studies (published in 2000 or after), and studies conducted in Australia and other English-speaking countries.

We found that most definitions agree that QoL refers to subjective evaluations of one’s life across different domains. However, there was no consensus as to which domains constitute QoL. Based on the definitions we found, the World Health Organization’s (WHO) definition of QoL was identified as the most comprehensive and relevant for the purposes of this study:

“[An] individual’s perception of their position in life in the context of the culture and value systems in which they live and in relation to their goals, expectations, standards, and concerns. It is a broad-ranging concept affected in a complex way by the persons’ physical health, psychological state, level of independence, social relationships and their relationship to salient features of their environment.” [24]

The literature review formed the basis of consultation with study partners including PLHIV peer organisations, allowing for articulation of the four key domains. These conceptual domains were then ranked by a panel of experts from PLHIV peer organisations according to their conceptual accuracy, as well as relevance and usefulness to health and community programs. The four identified domains were ranked in the following order: 1) psychological domain (including mood, coping, hope and fear of the future, and self-worth); 2) social domain (including personal and social life, belonging, support, and social stigma); 3) health domain (including perception of one’s health, health-related concerns, energy, and HIV management); and 4) functional domain (including ability to live a “normal” life, independence, meaningful occupation, and satisfactory standard of living). These domains closely resemble the WHO conceptualisation of QoL with the functional domain effectively incorporating a level of independence and relationship to the environment.

### Development of items

The initial pool of items was developed by the first four authors of this article. A large number of candidate items were developed (over 100 items across the four domains). The items were pre-tested for face and content validity through an online survey with a panel of experts from PLHIV peer organisations (*n* = 13), and other HIV experts and stakeholders (*n* = 5). The panel members were asked to assess the relevance of the proposed items and whether they thought items should be retained for the next stage of the study. Based on the input from the panel the pool of items was reduced to 64.

### Validation study

#### Study design

To develop and test PozQoL psychometrically, we conducted an online survey of adult Australians living with HIV, including a one-month follow-up survey to examine test-retest reliability. The study had the following aims: 1) to identify scale items for inclusion in PozQoL and test construct validity; 2) to assess the internal and test-retest reliability of PozQoL; 3) to examine the concurrent and convergent validity of PozQoL against other established QoL measures, and measures of health, psychological and social wellbeing.

#### Data collection

The baseline survey was hosted online between 22 March and 31 May 2017. Participants were invited to complete a follow-up survey 1 month after they completed the initial survey. This included a subset of questions from the baseline survey that enabled assessment of the test-retest reliability of PozQoL. The baseline survey was advertised through PLHIV community organisations and online platforms including Facebook, the Facebook page of the Institute of Many (an online community of over 1000 Australian PLHIV), and Grindr (dating app for gay and bisexual men). Additionally, hard copies of the survey were distributed through PLHIV community organisations in Victoria. As an incentive, participants had the option to enter into a prize draw to win a tablet computer. Participants were asked to confirm that they consent to use their partial data at two points in the survey. In addition, by the end of the survey participants were given the option of withdrawing all their data by ticking an appropriate box.

Those who completed the baseline survey were asked to provide their email address if they wished to complete the follow-up survey. These participants were contacted approximately 1 month after they completed the baseline. The follow-up survey included the pilot PozQoL scale and basic demographic information from the baseline survey. In addition, to control for potential changes in QoL unrelated to HIV, participants were asked to list any major changes or events that occurred in the month leading to the follow-up survey. In both the baseline and follow-up surveys, participants were asked to provide their month of birth and the initials of their name, which enabled us to generate a unique participant code for matching baseline and follow-up responses while protecting anonymity. It took participants 33 min on average to complete the baseline survey and 15 min to complete the follow-up survey.

### Survey measures

#### PozQoL

The pilot version of PozQoL including 64 items was used (see Table 2 for examples of item wording). Participants scored their response to each item on a 5-point scale (1 - *not at all*, 2 - *slightly*, 3 - *moderately*, 4 - *very*, 5 - *extremely*). A 5-point scale was chosen to balance sufficient gradation in responses with psychometric quality.

#### Other QoL measures

For the assessment of concurrent validity, we selected two established QoL measures which were previously validated on PLHIV samples and were freely available (thus could potentially be used by the community organisations). We chose one generic and one HIV-specific QoL measure to verify how well PozQoL corresponds both with broader and more specific conceptualizations of QoL.

##### Generic QoL

Medical Outcomes Study Short Form-36 (SF-36) [25] is a widely used generic measure of HRQoL, validated for use with PLHIV [26]. The scale incorporates 36 items grouped into eight domains, including physical and mental health (e.g.*, How much of the time during the past 4 weeks have you been a very nervous person?, I am as healthy as anybody I know*)*,* as well as functional wellbeing and role impairment (e.g.*, During the past 4 weeks, how much did pain interfere with your normal work?).* Response format and recall period vary between the items (for most items participants are asked to report on their QoL in 4 weeks prior to the survey). Responses were recoded in line with the RAND 36-Item Health Survey scoring algorithm [27] so that higher scores indicated a more favourable health state. Subsequently, summary scores for the physical (PCS) and mental component (MCS) were computed and normalized in line with Australian population scoring coefficients [28]. Correlation between the two composite scores was *r* (378) = .88, *p* < .001.

##### HIV-specific QoL

Functional Assessment of Human Immunodeficiency Virus Infection (FAHI; [18] Version 4) is an HIV-specific validated measure of HRQoL. The scale includes 47 items grouped into five domains: physical wellbeing, functional and global wellbeing, emotional wellbeing/living with HIV, social wellbeing, and cognitive functioning. Participants rated to what extent each statement *(*e.g.*, I feel fatigued)* described their experiences in the 7 days prior to the survey on a 1 *(not at all)* to 5 *(very much)* scale. Responses were recoded so that higher scores indicated better QoL (α = .97).

#### Mental health and wellbeing measures

To determine convergent validity, we selected a number of measures assessing mental health and wellbeing:

##### Psychological distress

Kessler Psychological Distress Scale (K6) ([29] is used widely in Australia as a screening measure for depression and anxiety. Participants reported how often they felt in a particular way (e.g., ‘*restless or fidgety’*) in the 30 days prior to the survey on a 1 (*all the time*) to 5 (*none of the time*) scale (α = .93).

##### Wellbeing

The Short Warwick-Edinburgh Mental Wellbeing Scale (S-WEMWBS) [30] was used to assess positive mental health. Participants indicated how much each of the seven items included in the scale (e.g., *I’ve been feeling useful*) described their experiences during 2 weeks prior to the survey, on a 1 (*none of the time*) to 5 (*all of the time*) scale (α = .93).

##### Satisfaction with life

Satisfaction with Life Scale (SWLS) [31] was used. Participants indicated their agreement with five items, (e.g., *In most ways my life is close to my ideal*), on a 1 (*strongly disagree*) to 7 (*strongly agree*) scale (α = .92).

##### Resilience

The Brief Resilience Scale (BRS) [32] was used. Participants indicated their agreement with six items (e.g., *I tend to bounce back quickly after hard times*), on a 1 (*strongly disagree)* to 5 (*strongly agree*) scale (α = .91).

##### AIDS-related stigma

Internalized AIDS-Related Stigma Scale (IA-RSS) [33] was used. Participants indicated their agreement with six items (e.g., *Being HIV positive makes me feel dirty*) by choosing 1 (*agree*) or 2 (*disagree*); (α = .87).

##### Social support

Interpersonal Support Evaluation List [34]; ISEL-12 version [35] was used. Participants indicated to what extent each of the 12 statements (e.g., *I feel that there is no one I can share my most private worries and fears with*) was true of them, on a 1 (*definitely true*) to 4 (*definitely false*) scale. Responses were recoded so that higher scores indicated stronger social support (α = .93).

### Statistical analysis

Exploratory Factor Analysis (EFA) was used to explore the underlying factor structure and reduce the number of items. The final model comprising selected items was tested in a Confirmatory Factor Analysis (CFA) usin*g* a range of goodness-of-fit measures. Prior to analyses, the sample was randomly divided into two subsamples for conducting the EFA and CFA, respectively. Due to the iterative nature of the steps in these analyses, the detailed methods are described in the results section. Inter-item reliability for the final scale and subscales was assessed with Cronbach’s alpha. Temporal stability (test-retest reliability) of the scale was assessed among the participants who completed the follow-up sample, with intraclass correlation coefficients (ICCs). Descriptive statistics including means, standard deviations, and skewness coefficients of the scale were also assessed. Concurrent and convergent validity was assessed by analysing Pearson bi-variate correlation coefficients between the scale and other QoL, mental health, and wellbeing measures.

## Results

### Sample profile

A total of 465 participants living with HIV in Australia and aged 18 years and older (*M*_age_ = 47.26, *SD* = 11.98) completed the baseline survey. The sample consisted of 378 men and 14 women (73 participants either did not answer the question or indicated some other gender). The majority of men identified as gay (88.4%). Participants were from all Australian states and territories. Just under half were university educated (45.6%). The majority lived in urban or suburban areas (78.9%) and were Anglo-Celtic or European (72.3%) (see Table 1 for a detailed description of the analysed sample). Of this sample, 149 were contacted for the follow-up survey, with 80 (53.7%) completing the follow-up. Of this group, 51 participants listed no major changes or events occurring in their lives in the time between the two surveys and were included in the follow-up sample.

**Table 1:** Sample profile (*n* = 465)

			Number	Percent
Age				
18–34			34	7.3
35–49			157	33.8
50–64			208	44.7
65+			66	14.2
Education				
Secondary/non-university tertiary or below			252	54.3
University educated			212	45.6
Residential location				
Inner city			290	62.4
Suburban			77	16.6
Regional/rural			98	21.1
Year first tested positive for HIV				
1980–1995			105	22.6
1996–2009			178	38.3
2010–2017			172	37.0
Year started treatment				
1980–1995			50	10.8
1996–2009			174	37.4
2010–2017			220	47.3
Treatment status				
Currently taking ART			447	96.1
Not taking ART			17	3.7
Most recent viral load test				
Undetectable			432	92.9
Detectable			25	5.4
	Mean	(SD)	Possible range	
SF-36 PCS	44.30	(11.68)	N(50, 10)	
SF-36 MCS	41.14	(13.42)	N(50, 10)	
FAHI	3.63	(0.82)	1–5	
K6	3.82	(1.00)	1–5	
S-WEMWBS	3.41	(0.87)	1–5	
SWLS	4.13	(1.69)	1–7	
BRS	3.34	(0.93)	1–5	
IA-RSS	0.47	(0.37)	0–1	
ISEL-12	2.86	(0.77)	1–4	

SF-36 Medical Outcomes Study Short Form-36, PCS Physical Component Score, MCS Mental Component Score, FAHI Functional Assessment of Human Immunodeficiency Virus Infection, K6 Kessler Psychological Distress Scale, S-WEMWBS Short Warwick-Edinburgh Mental Wellbeing Scale, SWLS Satisfaction with Life Scale, BRS The Brief Resilience Scale, IA-RSS Internalized AIDS-Related Stigma Scale, ISEL-12 Interpersonal Support Evaluation List

### Preliminary analyses

Participants were excluded from the analysis if they had missing data on more than one PozQoL item (> 1.6% missing data). Prior to main analyses, the sample from the baseline study was split randomly into the EFA (*n* = 270) and CFA (*n* = 195) subsamples. Follow up comparisons did not reveal any significant differences between the two subsamples with regard to demographic characteristics, QoL, mental health or wellbeing. Analyses were performed with IBM SPSS Statistics and AMOS 24. The Keiser-Meyer-Olkin measure of sampling adequacy was above the recommended value of .60, KMO = .96, and Bartlett’s test of sphericity was significant: χ2 (2016) = 16,107.90, *p* < .001. We, therefore, proceeded with an EFA.

### EFA

Criteria for EFA were guided by recent theoretical and practical recommendations for scale development [36–39]. Both Mardia’s multivariate skewness and kurtosis tests [40], as well as the Doornik-Hansen test [41] rejected the null hypothesis of multivariate normality (all ps < .001). Principal axis factors extraction was therefore chosen over maximum likelihood extraction as the recommended factor extraction method for non-normally distributed data [39]. Because we expected that different domains of QoL would be inter-related, an oblique rotation (direct oblimin) was chosen, allowing the factors to be correlated [37]. Upon obtaining the initial solution, items with low communalities (< .50; [42]), low primary factor loadings (< .50; [37]), and high cross-loadings (≥ .32; [43]) were excluded in an iterative process. The subsequent EFAs were computed each time items were deleted (11 iterations were used). Thirty-one items were retained in the final solution (see Table 2 for standardized factor loadings and communalities for all retained items). Analysis revealed four factors (based on the scree test, eigenvalues > 1; and rejection of factors with fewer than four items [37, 43], which explained 71.8% of the variance. The rotated factor loadings were 14.36 (factor 1), 9.03 (factor 2), 10.57 (factor 3), and 9.57 (factor 4). Correlations between the factors ranged from .38 to .65.

**Table 2:** Exploratory factor analysis structure coefficients and extracted communalities for items assessed for the PozQoL scale

Scale item	Communalities (h^2^)	Factor loadings (standardized coefficients)			
		1	2	3	4
Having HIV prevents me from doing what I want to do	0.78	0.88			
Managing HIV disrupts my life	0.74	0.87			
Managing HIV gets in the way of my everyday life	0.69	0.84			
**I feel that HIV prevents me from doing as much as I would like**	**0.77**	**0.83**			
Having HIV prevents me from doing things that are important to me	0.80	0.84			
Managing my health disrupts my life	0.72	0.83			
**Managing HIV wears me out**	**0.70**	**0.82**			
I feel that having HIV limits my capacity to fulfil the different roles I have in life	0.76	0.79			
Managing my health gets in the way of my everyday life	0.65	0.76			
**Having HIV limits my opportunities in life**	**0.73**	**0.75**			
I feel that HIV prevents me from living a normal life	0.81	0.74			
Managing HIV is a difficult thing in my life	0.64	0.65			
I feel that HIV limits my social life	0.67	0.63			
I feel that I am struggling with my health	0.62	0.51			
I worry about what people will think of me when they find out I have HIV	0.85		−0.89		
I worry about other people finding out I have HIV	0.74		−0.86		
I worry that people will treat me unfairly once they learn I have HIV	0.76		− 0.79		
**I am afraid that people may reject me when they learn I have HIV**	**0.69**		**−0.77**		
I feel ashamed of living with HIV	0.69		−0.73		
Having HIV makes me feel inferior to other people	0.63		−0.63		
**I feel that HIV limits my personal relationships**	0.60		**−0.55**		
I am happy with my life	0.80			0.93	
**I am enjoying life**	**0.82**			**0.93**	
**I feel in control of my life**	**0.73**			**0.79**	
I feel that I can deal with whatever comes my way	0.60			0.77	
**I am optimistic about my future**	**0.61**			**0.77**	
**I feel good about myself as a person**	**0.68**			**0.77**	
**I worry about the impact of HIV on my health**	**0.83**				**0.80**
I worry about my health getting worse	0.76				0.79
**I fear the health effects of HIV as I get older**	**0.70**				**0.76**
**I worry about my health**	**0.73**				**0.63**

Items in bold are included in the final scale

### Further reduction in the number of items

Results of the EFA were then used as guidance in the further reduction in the number of items. To preserve the recommended acceptable minimum of three items per factor [44], our aim was to narrow down the pool of items to 12 in the final scale. This decision was driven by concern for parsimony and the ease of administering PozQoL. Item reduction followed standard psychometric criteria for item analysis: mean, standard deviation, cumulated proportion in the two extreme categories, corrected item-total, and between-item correlations were used to identify items exhibiting floor and ceiling effects (> 75%), low item-total correlation (< .50) or redundancy (between-item correlations > .80). Alongside the psychometric criteria, we took into account content validity of the items, drawing on the research literature and insights from the participation of PLHIV reflected in the conceptual model. Thus, the final pool included items that displayed good statistical qualities and met the conceptual requirements best.

In consultation with the PLHIV peer organisations, we identified that the criteria employed in the EFA had omitted one important component of the social domain from the conceptual model, namely ‘belonging’. We, therefore, retrieved an item from the initial pool*,* which had good face validity (*I lack a sense of belonging with people around me)* and fulfilled the applied psychometric criteria. Similarly, although our initial goal was to retain three items per domain, in consultation with the PLHIV peer organisations, we retained the four strongest items for the psychological subscale to maintain content validity for this domain. The final scale, therefore, comprised 13 items.

### CFA

**Fig. 1:**
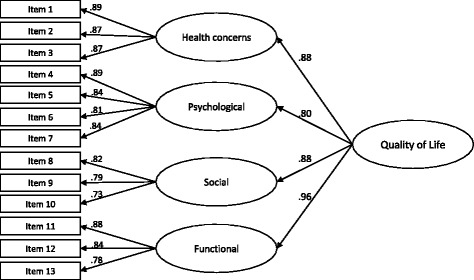
CFA of 13 items from the PozQoL scale

We tested the 13-item scale in a CFA, on the randomly selected CFA subsample (*n* = 195). The analysis was performed with the maximum likelihood procedures on the covariance matrix. Items were constrained to each load on one factor. Goodness-of-fit was assessed with a model chi-square analysis, the comparative fit index (CFI), standardized root-mean-square residual (SRMR), and the root-mean-square error of approximation (RMSEA). The following guidelines were used for optimal fit (Hu & Bentler, 1999): *p* > .05 for the model chi-square; CFI > .95; SRMR < .08; RMSEA < .06. The four-factor solution achieved excellent levels of fit: Χ_(61)_^2^ = 74.42, *p* = .116; CFI = .992, SRMR = .036, RMSEA = .034 [.000, .059]. Figure 1 shows results from the CFA, including standardized pattern coefficients for each item, all of which were above .70 (values ranged from 0.73 to 0.89).

### Internal consistency and test-retest reliability

Thirteen items retained in the final PozQoL scale are displayed in Table 3. All corrected item-total correlations exceeded 0.60 (range = 0.65–0.80), all inter-item correlations were below 0.80 (average homogeneity index = 0.58; range = 0.40–0.79).

**Table 3:** The PozQoL scale

I. health concerns	
1. I worry about my health	
2. I worry about the impact of HIV on my health	
3. I fear the health effects of HIV as I get older	
II. psychological	
4. I am enjoying life	
5. I feel in control of my life	
6. I am optimistic about my future	
7. I feel good about myself as a person	
III. social	
8. I feel that HIV limits my personal relationships	
9. I lack a sense of belonging with people around me 10. I am afraid that people may reject me when they learn I have HIV	
IV. functional	
11. I feel that HIV prevents me from doing as much as I would like	
12. Having HIV limits my opportunities in life	
13. Managing HIV wears me out	

“This survey is intended for people living with HIV. We would like to ask you about your health, relationships, life satisfaction, and wellbeing. Please indicate how much the following statements apply to you on a scale from 1 – not at all to 5 – extremely.” 1 – not at all 2 – slightly 3 – moderately 4 – very 5 – extremely

Inter-item reliability (assessed with Cronbach’s alpha) for the PozQoL was α = 0.95 [95% CI: (.93 to .96)], indicating excellent internal consistency. Reliabilities for particular subscales were as follows: α = 0.91 [95% CI: (.89 to .93)] - health concerns; α = 0.91 [95% CI: (.89 to .93)] – psychological; α = 0.82 [95% CI: (.77 to .86)] – social; and α = 0.87 [95% CI: (.84 to .90)] - functional.

Temporal stability (test-retest reliability) of the scale was assessed among the 51 participants who comprised the follow-up sample. Intra-class correlation coefficients (ICCs) were computed between baseline and follow-up scores based on a one-way random effects model. Scores on the PozQoL displayed high levels of stability, ICC = 0.95 [95% CI: (.92 to .97)]. Similarly, stable results were obtained on scores for all subscales: ICC = 0.91 [95% CI: (.85 to .95)] - health concerns; ICC = 0.85 [95% CI: (.74 to .92)] – psychological; ICC = 0.83 [95% CI: (.71 to .91)] – social; and ICC = 0.89 [95% CI: (.80 to .93)] - functional.

#### Score construction

Scoring PozQoL was a two-step process. First, all negatively-worded items were recoded so that higher scores for all items indicated better QoL. Subsequently, items were averaged to create the total score and scores for each subscale. The response range for each scale was 1–5. Items with missing responses were not taken into account when calculating the scores. (Only responses of participants who missed none or one item were used to compute the scores. Missing data for each participant varied between 0 and 7.7%; missing data for each item varied between 0 and 0.9%). Thus, the scores represented the average for items in the subscale that the respondent answered.

#### Scale properties of the PozQoL

Means and standard deviations were *M* = 3.43 [95% CI: (3.35 to 3.51)] for the total PozQoL score, *M* = 3.22 [95% CI: (3.12 to 3.33)] for the health concern subscale, *M* = 3.38 [95% CI: (3.29 to 3.47)] for the psychological subscale, *M* = 3.27 [95% CI: (3.16 to 3.37)] for the social subscale, and *M* = 3.88 [95% CI: (3.79 to 3.98)] for the functional subscale, respectively. Diagnostic plots, including histograms and a standardized normal probability plot, indicated that PozQoL scores were moderately skewed, with a skewness coefficient of −.53. Skewness coefficients for the subscales varied between −.27 and − .93, indicating either an approximate symmetry or a moderate skew.

### Concurrent and convergent validity

Table 4 displays Pearson bi-variate correlation coefficients between the PozQoL and other QoL, mental health, and wellbeing measures. Correlation coefficients of .30–.49, .50–.69, and > .70 were interpreted as small, moderate, and large, respectively.

**Table 4:** Correlations between the PozQoL scale and measures of QoL, mental health, and wellbeing

	PozQoL total score	PozQoL health concerns	PozQoL psychological	PozQoL social	PozQoL functional
	r (95% CI)				
Quality of Life					
SF-36 PCS	.68 (.62–.74)	.54 (.46–.62)	.65 (.59–.71)	.45 (.36–.54)	.66 (.59–.72)
SF-36 MCS	.77 (.72–.81)	.57 (.48–.64)	.78 (.72–.82)	.57 (.48–.64)	.69 (.63–.74)
FAHI	.87 (.84–.90)	.69 (.63–.74)	.79 (.74–.83)	.72 (.67–.77)	.77 (.73–.82)
Mental health and wellbeing					
K6	.75 (.69–.80)	.55 (.47–.63)	.72 (.65–.78)	.60 (.52–.66)	.66 (.59–.72)
S-WEMWBS	.76 (.71–.81)	.56 (.48–.64)	.77 (.71–.82)	.61 (.53–.67)	.63 (.57–.69)
SWLS	.73 (.68–.78)	.53 (.45–.60)	.74 (.67–.79)	.57 (.50–.64)	.63 (.57–.69)
BRS	.68 (.61–.74)	.47 (.37–.56)	.71 (.66–.76)	.52 (.44–.60)	.57 (.49–.65)
IA-RSS	−.65 (−.70;-59)	−.49 (−.57;-.41)	−.47 (−.55;-.39)	−.71 (−.75;-.66)	−.54 (−.61;-.46)
ISEL-12	.59 (.51–.66)	.40 (.32–.49)	.54 (.45–.62)	.56 (.48–.63)	.49 (.41–.57)

*Note.* All correlations are significant at the *p* < .001 level; *N* varied between 373 and 407 due to missing data; *SF-36* Medical Outcomes Study Short Form-36, *PCS* Physical Component Score, *MCS* Mental Component Score, *FAHI* Functional Assessment of Human Immunodeficiency Virus Infection, *K6* Kessler Psychological Distress Scale, *S-WEMWBS* Short Warwick-Edinburgh Mental Wellbeing Scale, *SWLS* Satisfaction with Life Scale, *BRS* The Brief Resilience Scale, *IA-RSS* Internalized AIDS-Related Stigma Scale, *ISEL-12* Interpersonal Support Evaluation List

Concurrent validity was determined by assessing correlation coefficients between PozQoL and other measures of QoL. We expected that PozQoL would correlate positively both with a generic and an HIV-specific measure of QoL. Given that PozQoL was designed specifically for PLHIV, we expected the relationship with the latter to be stronger. The total score for PozQoL displayed a significant moderate correlation with the physical component of SF-36 and a strong correlation with the mental component of SF-36.

Similarly, PozQol displayed a strong correlation with FAHI, indicating a high level of agreement between the measured concepts. As expected, the correlation between PozQoL and FAHI was stronger than the correlation with the SF-36 composite scores (*z* = 7.10, *p* < .001 for PCS and *z* = 4.41, *p* < .001 for MCS, respectively).

Convergent validity was assessed with correlation coefficients between PozQoL and other measures of mental health and wellbeing. The total score for PozQoL displayed significant moderate to strong correlations with all measures.

## Discussion

Community and health services in the HIV sector expressed a need for an empirically validated instrument that measures the impact of programs on QoL among PLHIV. The development of a short easy to use instrument could transform the way HIV organisations, governments, and other stakeholders evaluate clinical and support programs, allowing these to be more firmly evidence-based and consistent across the sector.

In this study, we developed and tested a brief 13-item PozQoL scale assessing QoL among PLHIV. PozQoL displayed excellent construct validity and very good reliability, including consistency and temporal stability. The factor structure of the PozQoL scale is consistent with the conceptualization of QoL proposed by the World Health Organization [24] including four key factors: 1) health concerns; 2) psychological; 3) social; and 4) functional domains.

An assessment of concurrent and convergent validity indicated a significant overlap between PozQoL and two other established measures of QoL, and consistent correlations with other measures of mental health and wellbeing. Thus, PozQoL may be considered a shorter alternative to the use of multiple larger scales. The active partnership with PLHIV peer-led organisations enabled the study to ensure the research rigour was complemented by practical and conceptual considerations and contributed a deeper understanding of the complexity of the experience of PLHIV.

Based on feedback from stakeholders we believe the PozQoL scale may be useful in several ways, including evaluation of health and support programs for PLHIV, and as an indicator or assessment of HRQoL of PLHIV in research studies. The brevity of PozQol will enable the inclusion of HRQol in broader surveys of PLHIV, and facilitate comparison of HRQoL between groups of PLHIV with different needs and experiences, or at different stages of the continuum of care (diagnosis, treatment uptake, treatment adherence, viral suppression). Being brief and freely available also means that PozQol can be used in the evaluation of programs designed to improve QoL and treatment maintenance for PLHIV. This includes the capacity to monitor total and individual domain scores, particularly when a program may be focused on one or two of the domains. Incorporating QoL into service evaluation which otherwise would not be able to include a large scale may support the targeting of programs and services to meet and sustain the 90, 90, 90 goals. An implementation trial of the PozQoL scale will be conducted across clinical, community and peer-led programs in 2018.

## Limitations

Although our results indicate that PozQoL is a valid measure of QoL among PLHIV, the current study had several limitations. While the sample was diverse, the majority were Anglo-Australian gay-identifying men. Although this demographic group constitutes the vast majority of PLHIV in Australia [45] and so was to be expected, future research may be needed with larger samples of other genders, sexualities, and cultural backgrounds to confirm its applicability and reliability. Similarly, only Australian participants were involved in the study. Future research may be needed to test the PozQoL scale among PLHIV in other English-speaking countries, and to assess its capacity to be translated into other languages.

The majority of the sample was collected online and through community organizations and networks. It is feasible that our sample was not entirely representative of the PLHIV population in Australia, particularly individuals not connected with HIV services and community organisations.

Further, due to the nature of our study, the final 13-item PozQoL scale was not tested on its own, but as part of the wider set of 64 items. Future studies are thus needed to confirm the validity of the PozQoL as a standalone measurement tool.

Given the development and validity trial was cross-sectional, we have yet to assess the PozQoL scale’s sensitivity to detecting changes in QoL over time. The study will be implementing a usability and sensitivity implementation trial in 2018 across a range of health and community services in Australia. The PozQoL will also be trialled in cross-sectional studies in the USA, which due to the nature of the epidemiology, will enable us to test validity of the scale in more gender-, sexually-, ethnically-, and language-diverse samples than in Australia. For the purpose of the studies the PozQoL will be translated into Spanish.

## Conclusion

The PozQoL scale has been tested in a diverse sample of adult PLHIV living in Australia, demonstrating very good reliability and validity, as well as the strength of meaningful involvement of people with HIV in research studies. It is recommended that the scale is implemented and field-tested across a range of community, support, and healthcare programs for PLHIV and in other countries. This will make a significant contribution to the social research on PLHIV and the evaluation of programs for PLHIV.

## Abbreviations

ART: Antiretroviral Treatment
CFA: Confirmatory Factor Analysis
CFI: Comparative Fit Index
EFA: Exploratory Factor Analysis
GIPA: The Greater Involvement of People Living with HIV
HRQoL: Health-Related Quality of Life
PLHIV: People living with HIV
QoL: Quality of Life
RMSEA: Root Mean Square Error of Approximation
UNAIDS: The Joint United Nations Programme on HIV and AIDS
WHO: World Health Organization

## References

[1] Günthard HF, Saag MS, Benson CA, Del Rio C, Eron JJ, Gallant JE, Hoy JF, Mugavero MJ, Sax PE, Thompson MA (2016). Antiretroviral drugs for treatment and prevention of HIV infection in adults: 2016 recommendations of the international antiviral society–USA panel. JAMA.

[2] UNAIDS (2014). 90–90-90 an ambitious treatment target to help end the AIDS epidemic.

[3] World Health Organization (2016). Global health sector strategy on HIV 2016–2021. Towards ending AIDS.

[4] Erdbeer G, Sabranski M, Sonntag I, Stoehr A, Horst H-A, Plettenberg A, Schewe K, Unger S, Stellbrink H-J, Fenske S (2014). Everything fine so far? Physical and mental health in HIV-infected patients with virological success and long-term exposure to antiretroviral therapy. J Int AIDS Soc.

[5] Katz IT, Ryu AE, Onuegbu AG, Psaros C, Weiser SD, Bangsberg DR, Tsai AC (2013). Impact of HIV-related stigma on treatment adherence: systematic review and meta-synthesis. J Int AIDS Soc.

[6] Maiese EM, Johnson PT, Bancroft T, Goolsby Hunter A, Wu AW (2016). Quality of life of HIV-infected patients who switch antiretroviral medication due to side effects or other reasons. Curr Med Res Opin.

[7] Miners A, Phillips A, Kreif N, Rodger A, Speakman A, Fisher M, Anderson J, Collins S, Hart G, Sherr L (2014). Health-related quality-of-life of people with HIV in the era of combination antiretroviral treatment: a cross-sectional comparison with the general population. Lancet HIV.

[8] Lazarus JV, Safreed-Harmon K, Barton SE, Costagliola D, Dedes N, del Amo Valero J, Gatell JM, Baptista-Leite R, Mendão L, Porter K (2016). Beyond viral suppression of HIV–the new quality of life frontier. BMC Med.

[9] Australian Government (2014). Seventh national HIV strategy 2014–2017.

[10] Moons P, Budts W, De Geest S (2006). Critique on the conceptualisation of quality of life: a review and evaluation of different conceptual approaches. Int J Nurs Stud.

[11] Engler K, Lessard D, Lebouché B (2017). A review of HIV-specific patient-reported outcome measures. Patient.

[12] Duracinsky M, Herrmann S, Berzins B, Armstrong AR, Kohli R, Le Coeur S, Diouf A, Fournier I, Schechter M, Chassany O (2012). The development of PROQOL-HIV: an international instrument to assess the health-related quality of life of persons living with HIV/AIDS. J Acquir Immune Defic Syndr.

[13] Oberjé EJ, Dima AL, van Hulzen AG, Prins JM, de Bruin M (2015). Looking beyond health-related quality of life: predictors of subjective well-being among people living with HIV in the Netherlands. AIDS Behav.

[14] O'Brien KK, Bayoumi AM, Strike C, Young NL, King K, Davis AM (2010). How do existing HIV-specific instruments measure up? Evaluating the ability of instruments to describe disability experienced by adults living with HIV. Health Qual Life Outcomes.

[15] Horsman J, Furlong W, Feeny D, Torrance G (2003). The health utilities index (HUI®): concepts, measurement properties and applications. Health Qual Life Outcomes.

[16] Herdman M, Gudex C, Lloyd A, Janssen M, Kind P, Parkin D, Bonsel G, Badia X (2011). Development and preliminary testing of the new five-level version of EQ-5D (EQ-5D-5L). Qual Life Res.

[17] Wu A, Revicki D, Jacobson D, Malitz F (1997). Evidence for reliability, validity and usefulness of the medical outcomes study HIV health survey (MOS-HIV). Qual Life Res.

[18] Peterman A, Cella D, Mo F, McCain N (1997). Psychometric validation of the revised functional assessment of human immunodeficiency virus infection (FAHI) quality of life instrument. Qual Life Res.

[19] Schag C, Ganz PA, Kahn B, Petersen L (1992). Assessing the needs and quality of life of patients with HIV infection: development of theHIVOverview of problems-EvaluationSystem (HOPES). Qual Life Res.

[20] Ware Jr JE, Kosinski M, Keller SD. A 12-Item Short-Form Health Survey: construction of scales and preliminary tests of reliability and validity. Medical care. 1996;34(3):220–33. 10.1097/00005650-199603000-00003 8628042

[21] Hawthorne G, Richardson J, Osborne R (1999). The assessment of quality of life (AQoL) instrument: a psychometric measure of health-related quality of life. Qual Life Res.

[22] Schmidt S, Mühlan H, Power M (2005). The EUROHIS-QOL 8-item index: psychometric results of a cross-cultural field study. Eur J Public Health.

[23] UNAIDS (2007). The greater involvement of people living with HIV (GIPA): policy brief.

[24] World Health Organization (1997). WHOQOL: measuring quality of life.

[25] Brazier JE, Harper R, Jones N, O'cathain A, Thomas K, Usherwood T, Westlake L (1992). Validating the SF-36 health survey questionnaire: new outcome measure for primary care. BMJ.

[26] Wu AW, Hays RD, Kelly S, Malitz F, Bozzette SA (1997). Applications of the medical outcomes study health-related quality of life measures in HIV/AIDS. Qual Life Res.

[27] Hays RD, Sherbourne CD, Mazel RM (1993). The rand 36-item health survey 1.0. Health Econ.

[28] Tucker G, Adams R, Wilson D (2010). New Australian population scoring coefficients for the old version of the SF-36 and SF-12 health status questionnaires. Qual Life Res.

[29] Kessler RC, Andrews G, Colpe LJ, Hiripi E, Mroczek DK, Normand S-L, Walters EE, Zaslavsky AM (2002). Short screening scales to monitor population prevalences and trends in non-specific psychological distress. Psychol Med.

[30] Stewart-Brown S, Tennant A, Tennant R, Platt S, Parkinson J, Weich S (2009). Internal construct validity of the Warwick-Edinburgh mental well-being scale (WEMWBS): a Rasch analysis using data from the Scottish health education population survey. Health Qual Life Outcomes.

[31] Diener E, Emmons RA, Larsen RJ, Griffin S (1985). The satisfaction with life scale. J Pers Assess.

[32] Smith BW, Dalen J, Wiggins K, Tooley E, Christopher P, Bernard J (2008). The brief resilience scale: assessing the ability to bounce back. Int J Behav Med.

[33] Kalichman SC, Simbayi LC, Cloete A, Mthembu PP, Mkhonta RN, Ginindza T (2009). Measuring AIDS stigmas in people living with HIV/AIDS: the internalized AIDS-related stigma scale. AIDS Care.

[34] Cohen S, Syme SL (1985). Issues in the study and application of social support. Soc Support Health.

[35] Merz EL, Roesch SC, Malcarne VL, Penedo FJ, Llabre MM, Weitzman OB, Navas-Nacher EL, Perreira KM, Gonzalez F, Ponguta LA (2014). Validation of interpersonal support evaluation list-12 (ISEL-12) scores among English-and Spanish-speaking Hispanics/Latinos from the HCHS/SOL sociocultural ancillary study. Psychol Assess.

[36] Cabrera-Nguyen P (2010). Author guidelines for reporting scale development and validation results in the journal of the Society for Social Work and Research. J Soc Soc Work Res.

[37] Costello AB, Osborne JW (2005). Best practices in exploratory factor analysis: four recommendations for getting the most from your analysis. Pract Assess Res Eval.

[38] Fabrigar LR, Wegener DT, MacCallum RC, Strahan EJ (1999). Evaluating the use of exploratory factor analysis in psychological research. Psychol Methods.

[39] Worthington RL, Whittaker TA (2006). Scale development research: a content analysis and recommendations for best practices. Couns Psychol.

[40] Mardia KV (1970). Measures of multivariate skewness and kurtosis with applications. Biometrika.

[41] Doornik JA, Hansen H (2008). An omnibus test for univariate and multivariate normality. Oxf Bull Econ Stat.

[42] Velicer WF, Fava JL (1998). Affects of variable and subject sampling on factor pattern recovery. Psychol Methods.

[43] Tabachnick BG, Fidell LS, Osterlind SJ (2001). Using multivariate statistics.

[44] Brown TA. Confirmatory factor analysis for applied research. New York: Guilford Publications; 2014.

[45] Kirby Institute (2016). HIV, viral hepatitis and sexually transmissible infections in Australia annual surveillance report 2016.

